# Calculation of the Absorbed Electron Energy 3D Distribution by the Monte Carlo Method, Presentation of the Proximity Function by Three Parameters α, β, η and Comparison with the Experiment

**DOI:** 10.3390/ma15113888

**Published:** 2022-05-30

**Authors:** Alexander A. Svintsov, Maxim A. Knyazev, Sergey I. Zaitsev

**Affiliations:** Institute of Microelectronics Technology and High Purity Materials, Russian Academy of Sciences, Chernogolovka, St. Academician Osipyan, 6, 142432 Moscow, Russia; svintsov@iptm.ru (A.A.S.); maleksak@iptm.ru (M.A.K.)

**Keywords:** electron-beam lithography, Monte Carlo method, proximity function, electrons scattering

## Abstract

The paper presents a program for simulating electron scattering in layered materials ***ProxyFn***. Calculations show that the absorbed energy density is three-dimensional, while the contribution of the forward-scattered electrons is better described by a power function rather than the commonly used Gaussian. It is shown that for the practical correction of the proximity effect, it is possible, nevertheless, to use the classical two-dimensional proximity function containing three parameters: α, β, η. A method for determining the parameters α, β, η from three-dimensional calculations based on MC simulation and development consideration is proposed. A good agreement of the obtained parameters and experimental data for various substrates and electron energies is shown. Thus, a method for calculating the parameters of the classical proximity function for arbitrary layered substrates based on the Monte Carlo simulation has been developed.

## 1. Introduction

One of the most common methods for creating micro- and nanostructures in microelectronics is electron-beam lithography (EBL). Although EBL is less productive than photolithography, it turns out to be very convenient to create small structures consisting of elements of very different sizes. This makes it in demand when creating micro- and nano-objects for scientific research. Such structures can be used for studies of superconductivity [1,2], X-ray radiation [3,4,5], electrophysical properties of various materials (for example, graphene [6,7]) and in many other areas of physics.

One of the features of EBL is the effect of electrons back-scattered in the substrate on the electronic resist. In this case, they carry out additional exposure of the electron resist on an area usually much larger than the size of the primary electron beam. This effect is commonly called the “proximity effect” [8]. Taking into account the influence and correction of the “proximity effect” on the dose absorbed by the electronic resist allows increasing the accuracy of electron lithography, shortens the time to fabricate structures and increases the yield of a suitable product, since it reduces the sensitivity of lithography to random errors. To correct the influence of the “proximity effect” when calculating the exposure dose, the proximity function (PF) is used, i.e., the distribution of the absorbed energy in the electron resist during electron-beam scattering. 

The classical PF *I* (*x,y*) consists of two Gaussians [8], does not change over the depth of the resist film *z*, is dimensionless and is normalized to unity [9,10]. It is written in the following form:

(1) $$I\left(x,y,\alpha ,\beta ,\eta \right)=\frac{\exp\left(-{\left(\frac{r}{\alpha }\right)}^{2}\right)}{\pi \alpha^{2}\left(1+\eta \right)}+\eta \frac{\exp\left(-{\left(\frac{r}{\beta }\right)}^{2}\right)}{\pi \beta^{2}\left(1+\eta \right)}$$

where *r^2^ = x^2^ + y^2^*, η is the ratio of the total energy left by the reflected electrons to the energy of the forward-scattered electrons. The first Gaussian in (1) describes the distribution of the energy left in the resist by the forward-scattered electrons (characterized by parameter α), and the second one describes the distribution of the energy left in the resist by the back-scattered electrons (characterized by parameter β) [11,12].

From (1), it follows that for the practical use of PF it is necessary to know the values of the parameters α, β, η. The experience of practical correction [10,13,14] and extensive simulations [15,16,17] show that the three parameters found in the experiment are in most cases quite enough to obtain the required lithography accuracy. 

**Figure 1:**
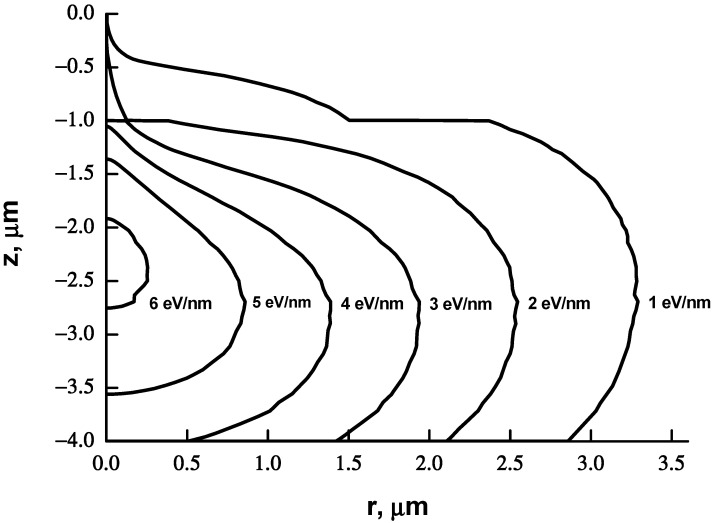
The results of the Monte Carlo calculation of the integral density of the absorbed energy *G*_r_(*r*, *z*). The values of the iso-levels of the density of the absorbed energy: 1, 2, 3, … eV/nm. The resist film thickness (PMMA) is 1 micron. The substrate is Si (300 μm). The initial energy of electrons is 25 keV. The number of trajectories considered is 200,000.

There is a large number of works devoted to calculations [12,18,19,20] and to experimental measurements of PF [21,22,23]. The MC method [24] has long been used for determining the parameters of different functions describing the proximity effect [12,18,19,20] by fitting the functions to the MC calculated spatial (3D) distribution of absorbed energy. Figure 1 shows the distribution of the absorbed energy density *G* (*r*, *z*) for a PMMA film of thickness H_0_ = 1um on a silicon substrate, calculated by the Monte Carlo method. It can be seen that *G* (*r*, *z*) varies in the thickness of the resist and is really a 3D function. For fitting the 2D proximity function to 3D data, usually a distribution of absorbed energy at the boundary resist–substrate (i.e., *G* (*r*, z = *H_0_*)) was used [12,18,19,20]. However, our experience has shown that consideration of the distribution only at the resist–substrate interface or averaging over the resist thickness does not allow one to obtain PF parameters that are in good agreement with the experimental values [21]. A particularly large discrepancy arises in the determination of the η parameter. Obviously, the cause is ignoring the process of development. Experimental methods for determining the PF parameters inevitably include development processes in the measurement procedure. When considering experimental methods, we prefer methods using resist development time (“vertical” methods) [21,22], over methods using measurements of the transverse dimensions of test features such as line widths or ring widths (e.g., [23]) (“horizontal” methods). Experimental determination of PF parameters is a laborious process and can take [21] several days or even weeks. The Monte Carlo calculation takes a few minutes. 

Therefore, the purpose of this work will be to develop an exclusively computational method for obtaining the parameters α, β, η of the 2D classical proximity function from the 3D absorbed energy density calculated by the Monte Carlo method with careful consideration of development. For comparison, experimental data α_e_, β_e_, η_e_ will be taken from [21].

## 2. Theory and Calculation

### 2.1. Calculation of Electron Scattering in Layered Materials by the Monte Carlo Method

We have developed an algorithm and implemented it in the ***ProxyFn*** program for the fast simulation of electron scattering in layered materials [25,26]. The structure of the algorithm is close to the one described in the work [24], but all expressions for the calculation are taken from the book by L. Reimer [27]. 

In the Monte Carlo simulation, it is assumed that electrons “move” in a straight line, continuously losing energy, until elastic scattering. A new direction of electron “moving” is played out using a screened Rutherford cross section [27]. The length of the straight segments is chosen randomly based on the total cross section of elastic scattering along the trajectory. Before scattering, in correspondence to the continuously slowing down approximation, the electron energy decreases, taking into account the length of the segment and the current value of stopping power (for details see [12,13,14,15,16,17,24]). 

In calculations, the sample is a layered structure consisting of an arbitrary number of layers and arbitrary materials. For convenience, the sample is automatically divided into cells by a grid. In the cells of the partition grid (*r*_i_, *z*_j_), the energy of electrons *E* (*r*_i_, *z*_j_) left by them when passing through these cells and the number of stopped electrons N (*r*_i_, *z*_j_) are remembered. Additionally, the coefficients of reflection and transmission of electrons from the entire sample are determined, as well as the coefficients of the absorption of electrons and energy in all layers. To start the calculation, it is necessary to know only the starting energy of electrons, film thickness, chemical formulas of materials and their density. For many elements and materials, the chemical composition and density can be selected from the built-in database.

To speed up the calculation, a cylindrically symmetric and nonuniform grid *r*_i_, *z*_j_ with center *r* = 0 on the beam axis is used. The *z* axis is perpendicular to the layers and directed from the outer boundary of the resist to the sample. *z* = 0, *r* = 0 is the point of entry of electrons into matter. The partition grid is set automatically.

The calculation does not consider the generation, scattering and absorption of secondary electrons, to which the fast electron gives up energy during deceleration. Although it is the secondary electrons with energies up to 50 eV that do all the “work” of breaking chemical bonds or forming bonds in resist molecules, their track length in materials does not exceed 10 nm [28] and can be taken into account by the convolution of the calculated absorbed energy density with the corresponding Gaussian. The Monte Carlo calculations also do not take into account the charging of dielectric layers, which can significantly change the trajectory of an electron. We believe that this problem can be effectively dealt with in electron lithography (for example, by applying a conductive film to the resist and grounding it, or by using a conductive resist). The PF calculation time for ten thousand trajectories takes several minutes.

### 2.2. Integral Proximity Function: Fitting of Absorbed Energy Distribution by Elementary Functions

It is not very difficult to implement the Monte Carlo algorithm for simulating electron trajectories. Difficulties arise when analyzing the calculation results and fitting the simulation results. After enumerating a large number of options, the following function was chosen to interpolate the density of the distribution of the electron absorbed energy *G(x,y,z)* in the sections *z = const*:

(2) $$G\left(x,y,z\right)=C_{\delta }\left(z\right)\delta \left(x\right)\delta \left(y\right)+\frac{C_{a}\left(z\right)}{{\left(1+{\left(\frac{r}{\alpha \left(z\right)}\right)}^{2}\right)}^{2}\pi \alpha^{2}\left(z\right)}+\frac{C_{b}\left(z\right)\exp\left(-{\left(\frac{r}{\beta \left(z\right)}\right)}^{2}\right)}{\pi \beta^{2}\left(z\right)}$$

where *r* is the distance to the beam axis. The first (delta-shaped) element in expression (2) describes the electrons of the primary beam, which have not experienced one scattering on atomic nuclei. The second and third elements in (2) describe (on a qualitative level) singly and multiply scattered electrons, respectively. The coefficient *C_δ_*(*z*) in the first approximation decreases exponentially with the penetration depth *z*, as

Cδ(z)=exp(−zLf)

where *L_f_* is the free length. It is inversely proportional to the total cross section of electron scattering in the resist and is equal to several tens of nanometers (about 80 nm for 25 keV electrons in PMMA). In the experiment, it is not easy to separate the first and second elements in expression (2) due to the fact that the initial electron beam is not delta-shaped.

Note that the fitting parameters *C_δ_*(*z*), *C*_a_(*z*), *C*_b_(*z*), α(*z*) and β(*z*) depend essentially on the depth *z* and, in this case, the relation α(*z*) << β(*z*) is fulfilled.

To search for the fitting parameters *C_δ_*(*z*), *C*_a_(*z*), *C*_b_(*z*), α(*z*) and β(*z*), it is convenient to use not the distribution density *G*(*x,y,z*) itself, but the integral density of the absorbed energy *G*_r_(*r*,*z*) obtained from the expression:

(3) $$G_{r}(r,z)=\int_{r}^{\infty }2\pi r^{\prime }dr^{\prime }G(r^{\prime },z)$$

Note that the integral density *G*_r_(*r*,*z*) can be given a physical meaning. Consider a special structure in the form of an infinite plane with a cut out circle of radius *r*. It turns out that exposure of such a structure with a single dose leads to the absorbed energy density *dE/dz* at the center of the circle just equal to *G*_r_(*r*,*z*). 

Using (2)–(3), we obtain for the three-dimensional proximity function:

(4) $$G_{r}(r,z)=C_{\delta }\left(z\right)\Theta \left(r\right)+\frac{C_{a}\left(z\right)}{1+{\left(\frac{r}{\alpha \left(z\right)}\right)}^{2}}+C_{b}\left(z\right)\exp\left(-{\left(\frac{r}{\beta \left(z\right)}\right)}^{2}\right)$$

here

$$\Theta (\rho )=\{\begin{array}{c} 1,\;\;\;\rho =0\;\\ 0,\;\;\;\rho >0 \end{array}$$

On the other hand, the integrated absorbed energy density *I_r_(x*,*y*) in the case of the classical proximity function (1) will be equal to:

(5) $$I_{r}\left(r,\alpha ,\beta ,\eta \right)=\int_{r}^{\infty }2\pi r’dr’I\left(r’,\alpha ,\beta ,\eta \right)=\frac{\exp\left(-{\left(\frac{r}{\alpha }\right)}^{2}\right)+\eta \exp\left(-{\left(\frac{r}{\beta }\right)}^{2}\right)}{1+\eta }$$

**Figure 2:**
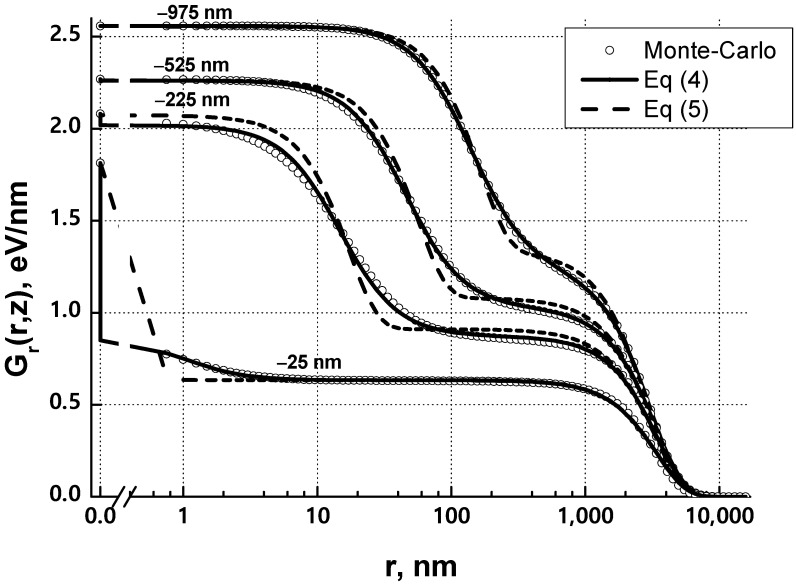
The results of fitting the integral density of the absorbed energy, calculated by the Monte Carlo method, by two different functions (4) and (5) for four cross sections in *z*: −25 nm, −225 nm, −525 nm and −975 nm. The resist film thickness (PMMA) is 1 micron. The substrate is Si (300 μm). The initial energy of electrons is 25 keV. The number of trajectories considered is 200,000.

Figure 1 and Figure 2 show an example of calculating the integrated absorbed energy density *G*_r_(*r*,*z*) and its approximation for a 1 μm thick PMMA e-beam resist film on a 300 μm thick silicon substrate at an initial electron energy of 25 keV. The number of trajectories considered is 200,000. The classical PF consisting of two Gaussians does not approximate *G*_r_(*r*,*z*) very well, and function (4) completes it almost ideally in all sections *z*.

Thus, the depth-dependent three-dimensional PF *G*(*r*,*z*) can, in principle, be used to correct the proximity effect in e-beam lithography. However, a three-dimensional PF has significantly more fitting parameters than a classical PF with only three fitting parameters (б, в, з). This leads to a complication of calculations. On the other hand, as mentioned, the practical use (to correct the “proximity effect”) of the classical PF leads to good results. Therefore, our next purpose is to determine the effective parameters of a two-dimensional PF from the three-dimensional Monte Carlo simulation results.

### 2.3. Fitting with Three Parameters: Analogue of Experiment

An experimental method for determining the classical PF *I*(*x*,*y*,α,β,η) (1) was proposed in [21]. The idea of the method is to search for such parameters (б, в, з) so that the test structure, consisting of elements of different sizes, after correcting the proximity effect (calculating the corrected dose based on the classical PF *I*(*x*, *y*, α, β, η)) and exposure the positive resist, will be revealed exactly to the substrate in the center of each element. This experimental method and the measured parameters б, в, з from [21] have been used for more than 20 years to correct the proximity effect in the NanoMaker software and hardware complex for electron-beam lithography (www.nanomaker.com, accessed on 26 May 2022) with consistently good results.

A similar method to search for the parameters of the classical PF *I*(*x*,*y*,α,β,η) is used in this work. As in the experimental method, the calculation of the exposure dose *T*(*x*,*y*) (electron density per unit area) is based on the classical PF *I*(*x*,*y*,α,β,η) from expression (1), but the simulation is performed instead of actual exposure and development. The absorbed dose (density of absorbed energy per unit volume) *D*(*x*,*y*,*z*) in the simulation is calculated based on the three-dimensional PF *G*(*x*,*y*,*z*) obtained by Monte Carlo from expression (2). For the dimensionless classical PF *I*(*x*,*y*,α,β,η), the distribution of the absorbed dose *D*(*x*,*y*) is as follows:

(6) $$D(x,y)/D_{0}=\iint I(x-x’,y-y’,\alpha ,\beta ,\eta )T(x’,y’)/T_{0}dx’dy’$$

The development of a positive e-beam resist is simulated in the approximation of isotropic, local etching [29,30]. Then, the development rate *V* can be written as follows:

(7) $$V/V_{0}={\left(D/D_{0}\right)}^{\gamma }$$

where γ is the contrast of the resist; and *D*_0_, *V*_0_ are the technological constants. For a positive e-beam resist, the sensitivity *T*_0_ is defined as the exposure dose at which an element with dimensions much larger than в is revealed in the center exactly to the substrate. 

A brief description of the approach presented in Appendix A is as follows. The proposed method consists in considering a number of circular elements of different sizes *R*. Exposing a circle with a uniform exposure dose results in an absorbed dose distribution with a maximum exactly at the center of any circular element at all resist depths *z.* From isotropic local etching theory [26,30], it follows that the development front reaches the substrate for the first time namely at the center of the round. Development times *T*_R_ and $T_{R}^{i}$ calculated for two different exposure models (for classical PF (5) and for three-dimensional PF (4)) are dependent on element radius *R*. Due to the simplicity of the elements, these times can be easily calculated by formulas. Further, such parameters of the classical PF б, в, з are searched with a special procedure that minimizes the objective function (9) using the ratio of the times *T_R_*/$T_{R}^{i}$.

To search for б, в, з, a set of 10 round elements of radius *R_n_* (*n* = 1,…,10) was used. The *R_n_* value varied from the minimum value α(*z*) to the maximum value β(*z*), 0 ≤ *z* ≤ *H*_0_, where α(*z*), β(*z*) are the interpolation parameters of the integrated density of the absorbed energy *G_r_*(*r*,*z*).

The Appendix (A4) shows that the exposure dose ratio

(8) $$\frac{T_{R}}{T_{R}^{i}}=\frac{{\left(\int_{0}^{H_{0}}\frac{dz}{G_{r}{}^{\gamma }\left(0,z\right)}\right)}^{1/\gamma }}{\left(1-I_{r}\left(R,\alpha ,\beta ,\eta \right)\right){\left(\int_{0}^{H_{0}}\frac{dz}{{\left(G_{r}\left(0,z\right)-G_{r}\left(R,z\right)\right)}^{\gamma }}\right)}^{1/\gamma }}$$

does not contain technological parameters *D*_0_, *V*_0,_ *t*_0_ and can be calculated relatively easily. The exposure dose ratios (8) were calculated (for the given parameters б, в, з) for the entire set of circles *R_n_*, and the objective function was composed from them.

(9) $$S={\sum_{n}\left(\frac{T_{R,n}^{}}{T_{R,n}^{i}}-1\right)}^{2}$$

Further, the values of the parameters α_s_, β_s_, η_s_ were determined by minimizing *S*(α, β, η). This method allows us to calculate quickly the parameters of a classical PF, depending on the thickness of the resist, the type of substrate (including the possible layered structure of the substrate), electron energy, etc. 

## 3. Results and Discussion

A comparison of the experimental parameters α_e_, β_e_, η_e_ and those obtained from the simulation based on Monte Carlo calculations α_s_, β_s_, η_s_ бs is necessary to verify the correctness of our chosen physical and mathematical models of electron scattering and resist development, as well as to verify the accuracy of calculations. It seems to us that a quantitative assessment of the accuracy of the Monte Carlo calculation (at least for thick resists) has not been performed before. 

The experimental data were taken from [21]. In the experimental method used in this work, each of the three parameters α_e_, β_e_, η_e_ was measured in a separate test. All tests used a PMMA electronic positive resist (chemical formula CH_2_C(CH_3_)(COOCH_3_), density 1190 kg/m^3^). In calculations based on the Monte Carlo method, all three parameters α_s_, β_s_, η_s_ were searched simultaneously. 

First, the data on the beam size б will be compared, and then the results of calculating в and з. The value of б depends on the energy, thickness and material of the resist and does not depend at all on the type of a substrate. In addition, the experimental value of б_e_ is influenced by the initial beam size б_0_, which is determined by the electron microscope setting (focusing, astigmatism) and beam jitter. In fact, the value of б_s_ obtained by the Monte Carlo method should be compared with parameter $\sqrt{\alpha_{e}^{2}-\alpha_{0}^{2}}$.

Table 1 shows a comparison of the size of the forward-scattered electron beam б, obtained from the б_e_ experiment (except the initial beam size б_0_) and calculated on the basis of the Monte Carlo method б_s_ for three energies *E* = 15, 25 and 35 keV and a set of resist thicknesses *H*_0_ = 100, 200, 500, 1000 and 1500 nm on a silicon substrate. The experimental data б_e_ were interpolated by the formula $\alpha_{e}^{2}=A_{E}H_{0}^{3}/E^{2}+\alpha_{0}^{2}$, where the constants *A_E_* and the initial beam dimensions б_0_ for different energies *E* were obtained from the experiment [21]. In the Monte Carlo calculation, the beam was assumed to be absolutely thin. Table 1 shows that the experimental data for б_e_ and the results of calculating б_s_ are in good agreement.

A comparison of the calculated (β_s_, η_s_) and experimental (β_e_, η_e_) for various substrates and accelerating voltages can be carried out using the data in Table 2, Table 3 and Table 4. For all cases, a positive PMMA resist 500 nm thick with a contrast γ = 3 was used. The values of α_s_, β_s_, η_s_ turned out to be stable with respect to the change in contrast, and hardly changed for г = 2.5, 3 or 4. For comparison, the experimental data for Si, GaAs, Al_2_O_3_ and mica from [21] were used; the data for Ge and C (diamond) substrates were specially measured for this work by the method [21]. The calculated values of β_s_ with an accuracy of ŷ10% coincided with the experimental values of β_e_, for з the accuracy was ŷ25%.

Note that the parameters в and з did not depend on focusing (if α_s_ << β_s_) and were determined only by the properties of the resist, substrate and the initial energy of electrons; therefore, they have a fundamental value.

## 4. Conclusions

The algorithm for the fast calculation of the absorbed energy density of electrons *G*(*r*) by the Monte Carlo method for layered materials was described. 

To interpolate the calculated absorbed energy density of electrons *G*(*r*), a fitting function (2) was proposed, which described well the distribution of the absorbed energy of electrons in layered materials depending on the distance to the center of the beam *r* and on the depth *z*. The power member describing the scattering of primary electrons seemed to be nontrivial and was considered for the first time. 

A numerical procedure was proposed that takes into account the development of the resist and makes it possible to replace the complex 3D distribution of the absorbed energy with a classical (two-dimensional) proximity function with three parameters б, в, з. 

The examples of calculating the parameters α_s_, β_s_, η_s_ of the proximity function were shown for different energies of electrons and substrates and their comparison with the experimental data α_e_, β_e_, η_e_. Calculations of в with an accuracy of ŷ10% coincided with the experiment; for з, the accuracy was ŷ25%.

Thus, it can be argued that the experimental confirmation of the accuracy of calculating PF by the Monte Carlo method and the procedure of interpolating PF with three parameters (б, в, з) has been obtained. 

## Figures and Tables

**Table 1:** Comparison of the parameters of the proximity function αs, (obtained by the method described above based on the Monte Carlo calculation) and αe, (calculated from the results of interpolation of experimental data [21]), for three values of the electron energy *E* and different thicknesses *H*_0_ of the PMMA resist. The substrate is Si.

*E*, keV	15		25		35	
*H*_0_, nm	α_e_, nm	α_s_, nm	α_e_, nm	α_s_, nm	α_e_, nm	α_s_, nm
100	6	4	3	2	2	1
200	16	12	10	6	7	5
500	64	56	39	30	28	20
1000	182	183	110	96	79	65
1500	334	342	202	196	145	130

**Table 2:** Comparison of the parameters of the proximity function obtained in the experiment β_e_, η_e_ and as a result of calculating β_s_, η_s_ for different energies of electrons *E* and Si and GaAs substrates. The PMMA resist (ERP-40) 0.5 μm thick.

Substrate	Si				GaAs			
Density	2330 kg/m^3^				5350 kg/m^3^			
*E*, keV	β_e_	β_s_	η_e_	η_s_	β_e_	β_s_	η_e_	η_s_
11	0.9	0.85	-	0.93	-	0.73	-	1.23
15	1.5	1.33	-	0.87	-	0.92	-	1.24
20	2.2	2.11	-	0.79	1.2	1.17	-	1.23
25	3.1	3.01	0.7	0.73	1.5	1.48	1.4	1.16
30	4	4.08	-	0.69	2	1.85		1.11
35	5.8	5.32	-	0.66	2.3	2.29	-	1.07
39	-	6.39	-	0.63	2.6	2.66	-	1.04

**Table 3:** Comparison of the parameters of the proximity function obtained in the experiment β_e_, η_e_ and as a result of calculating β_s_, η_s_ for different energies of electrons *E*, Al_2_O_3_ substrates and mica. The PMMA resist (ERP-40) 0.5 μm thick.

Substrate	Al_2_O_3_				KAl_2_Si_3_O_10_(OH)_2_ (mica)			
Density	3970 kg/m^3^				2850 kg/m^3^			
*E*, keV	β_e_	β_s_	η_e_	η_s_	β_e_	β_s_	η_e_	η_s_
11	-	0.76	-	0.76	0.75	0.81	-	0.81
15	1.	1.02	-	0.72	1.2	1.19	-	0.75
20	-	1.47	-	0.65	2	1.82	-	0.62
25	2	2.05	0.8	0.59	2.7	2.58	0.5	0.61
30	-	2.71	-	0.56	3.7	3.53	-	0.59
35	3.4	3.48	-	0.53	4.8	4.59	-	0.56
39	-	4.19	-	0.52	-	5.54	-	0.54

**Table 4:** Comparison of the parameters of the proximity function obtained in the experiment β_e_, η_e_ and as a result of calculating β_s_, η_s_ for different energies of electrons *E*, Ge substrates and diamond. The PMMA resist (ERP-40) 0.5 μm thick.

Substrate	Ge				С (Diamond)			
Density	5323 kg/m^3^				3500 kg/m^3^			
*E*, keV	β_e_	β_s_	η_e_	η_s_	β_e_	β_s_	η_e_	η_s_
11	-	0.73	-	1.26	0.7	0.79	-	0.51
15	0.7	0.92	-	1.28	1.0	1.08	-	0.41
20	1.1	1.15	-	1.24	1.6	1.61	-	0.33
25	1.4	1.46	1.1	1.18	2.1	2.23	0.4	0.3
30	1.8	1.84	-	1.12	2.6	2.96	-	0.29
35	2.5	2.27	-	1.08	3.6	3.87	-	0.26
39	-	2.67	-	1.06	-	4.63	-	0.25

## Data Availability

Data underlying the results presented in this paper are not publicly available at this time but may be obtained from the authors upon reasonable request.

## Appendix A

The ratio of exposure doses is found for classical and three-dimensional proximity functions. The density of absorbed energy (dose) in the center of a circle with the radius *R*, exposed with a constant dose *T* (density of electrons per unit area), is expressed through the integral proximity function:

(A1) $$D\left(R,z\right)=\left(G_{r}\left(0,z\right)-G_{r}\left(R,z\right)\right)\;T$$

For the classical dimensionless PF *I*(*x*,*y*,б,в,з), the absorbed energy distribution is written as follows:

(A2) $$\frac{D}{D_{0}}=\left(I_{r}\left(0,\alpha ,\beta ,\eta \right)-I_{r}\left(R,\alpha ,\beta ,\eta \right)\right)\frac{T}{T_{r}}=\left(1-I_{r}\left(R,\alpha ,\beta ,\eta \right)\right)\frac{T}{T_{0}}$$

In order for the resist to reveal exactly in the center of the circle to the substrate, the following condition should be held: *D*/*D*_0_ = 1.

Then, it follows from (A2) that the exposure dose *T_R_* should be equal to:

$$T_{R}=\frac{T_{0}}{1-I_{r}(R,\alpha ,\beta ,\eta )}$$

The “ideal” exposure dose $T_{R}^{i}$ is calculated using the three-dimensional PF. In the center of each element, the absorbed dose has a maximum on the *XY* plane in each section *z **=*** const; therefore, a positive electron resist is revealed vertically along the *z* axis in the center of the circle [30]. The absorbed dose *D_R_*(*z*) in the center of a circle with the radius *R_n_* exposed with a certain dose *T* is obtained from expression (A1). The time *t* for the development of a positive resist with a thickness *H_0_* to the substrate in the center of the circle is $t=\int_{0}^{H_{0}}\frac{dz}{V\left(z\right)}$. Then for a circle with the radius *R*:

(A3) $$t_{R}={\left(\frac{D_{0}}{T}\right)}^{\gamma }\int_{0}^{H}\frac{dz}{{\left(G_{r}\left(0,z\right)-G_{r}\left(R,z\right)\right)}^{\gamma }V_{0}}$$

The “ideal” exposure dose $T_{R}^{i}$ can be obtained from expression (A3), at which a circle with the radius *R* in the center is revealed to the bottom for a given development time *t*_0_:

$$T_{R}^{i}=\frac{D_{0}}{{\left(V_{0}t_{0}\right)}^{\frac{1}{\gamma }}}{\left(\int_{0}^{H_{0}}\frac{dz}{{\left(G_{r}\left(0,z\right)-G_{r}\left(R,z\right)\right)}^{\gamma }}\right)}^{\frac{1}{\gamma }}$$

The sensitivity of the positive resist *T*_0_ can also be calculated using this expression at *R* **= ∞**:

$$T_{0}=\frac{D_{0}}{{\left(V_{0}t_{0}\right)}^{\frac{1}{\gamma }}}{\left(\int_{0}^{H_{0}}\frac{dz}{G_{r}{}^{\gamma }\left(0,z\right)}\right)}^{\frac{1}{\gamma }}$$

As a result, the ratio of exposure doses *T_R_*/$T_{R}^{i}$ is obtained, which does not contain unknown technological parameters *D*_0_, *V*_0_ and *t*_0_:

(A4) $$\frac{T_{R}}{T_{R}^{i}}=\frac{{\left(\int_{0}^{H_{0}}\frac{dz}{G_{r}{}^{\gamma }\left(0,z\right)}\right)}^{1/\gamma }}{\left(1-I_{r}\left(R,\alpha ,\beta ,\eta \right)\right){\left(\int_{0}^{H_{0}}\frac{dz}{{\left(G_{r}\left(0,z\right)-G_{r}\left(R,z\right)\right)}^{\gamma }}\right)}^{1/\gamma }}$$
